# Tuberculous abdominal cocoon – a report of 6 cases and review of the Literature

**DOI:** 10.1186/1749-7922-1-18

**Published:** 2006-06-27

**Authors:** Robin Kaushik, RPS Punia, Harsh Mohan, Ashok K Attri

**Affiliations:** 1 Government Medical College & Hospital, Chandigarh, India

## Abstract

The abdominal cocoon is a rare cause of intestinal obstruction that is usually diagnosed at the time of laparotomy. It is usually of unknown origin, although at times, it may be seen secondary to a variety of conditions. Tuberculosis is an infrequently implicated cause of abdominal cocoon, and has only occasionally been reported previously in the Literature. This paper presents our experience with tubercular cocoon as a cause of intestinal obstruction, and discusses the surgical implications of the same.

## Background

Intestinal obstruction is a commonly encountered surgical emergency, and usually occurs secondary to intestinal adhesions, bands and obstructed herniae. However, at times, rare causes of intestinal obstruction may be encountered such as the '*abdominal cocoon*', also known as '*sclerosing encapsulating peritonitis*' (SEP), which is a rare condition that is characterized by the encasement of the small bowel by a fibrocollagenic cocoon like sac [1-4]. Although it was Foo et al (1978) who first named this as abdominal cocoon [1], the condition was first observed by Owtschinnikow in 1907 and was called as peritonitis chronica fibrosa incapsulata [2]. It is of two types – Primary or idiopathic, and secondary. The primary form of the disease is commoner, and has been classically described in young adolescent females from the tropical and subtropical countries. Here, the exact stimulus for the inflammatory reaction is not known, but some suggest that it may arise due to a subclinical primary viral peritonitis, as an immunological reaction to gynecological infections, or due to retrograde menstruation. However, since this condition has also been seen to affect males, premenopausal females and children, there seems to be little support for these theories [2-4].

Classically, SEP is of unknown etiology, but the secondary form of sclerosing peritonitis has been reported in association with practolol intake, chronic ambulatory peritoneal dialysis, ventriculoperitoneal and peritoneovenous shunts, sarcoidosis, SLE, liver cirrhosis, constrictive pericarditis being treated with propranolol, intraperitoneal instillation of drugs, leiomyomata of the uterus, endometriotic cyst or tumours of the ovary, and, recurrent peritonitis [2-9]. Only an occasional case of SEP occurring secondary to a tuberculous aetiology has been reported in medical literature [1,2,10]. We report a series of 6 cases of intestinal obstruction arising because of an abdominal cocoon of tuberculous etiology and review the relevant literature on this topic.

## Methods

Six patients presented to our hospital emergency over a period of 7 years (1999 to 2005) with acute intestinal obstruction that was diagnosed to be secondary to a tubercular abdominal cocoon on histopathology. The case files of these patients were reviewed retrospectively for the patient particulars, clinical presentation, operative findings and outcome. In addition, their histopathology slides were also reviewed to confirm the diagnosis of abdominal tuberculosis.

## Results

The average age of the patients was 21.8 years (range 9 years to 45 years); with 4 male and 2 female patients forming the study group (Table 1). All the patients presented with the classical signs and symptoms of acute intestinal obstruction, i.e. pain, vomiting, distension and constipation, varying over a duration of two months to 3 – 4 days prior to attending the hospital emergency. All the cases (except case number 4) were operated primarily at our hospital. The single case that was operated outside was referred to us in the first week after his surgery when he failed to 'open' up, and was re-explored by us for the non – resolution of his intestinal obstruction. Two cases had previous history of tuberculosis – one on treatment for abdominal, and the other, on treatment for pulmonary Koch's.

**Table 1:** Patient data

**S No.**	**Age Sex **	**Clinical presentation **	**Operative findings**	**Operative procedure**	**H/P findings**	**Outcome **
1	24 M	Intestinal obstruction	Cocoon Mesenteric abscess Tubercles	Adhesiolysis Drainage of abscess	Caseating epithelioid cell granulomas	Discharged Well on follow up
2	9 M	Intestinal obstruction	Cocoon Tubercles	Adhesiolysis	Caseating epithelioid cell granulomas	Enterocutaneous fistula Multiple reoperations Discharged after 4 months Well on follow up
3	23 F	Intestinal obstruction Epigastric mass	Cocoon Gangrene of terminal ileum Abscess	Adhesiolysis Resection Ileostomy and mucus fistula	Caseating epithelioid cell granulomas	Burst abdomen Enterocutaneous fistula Managed conservatively Discharged Well on follow up
4	45 M	Intestinal obstruction	Cocoon Interloop adhesions Tubercles	Adhesiolysis Resection and anastomosis	Caseating epithelioid cell granulomas	Reoperated for obstruction Died
5	17 M	Intestinal obstruction	Cocoon Interloop adhesions Tubercles	Adhesiolysis	Caseating epithelioid cell granulomas	Enterocutaneous fistula Reoperated and exteriorized Discharged Bowel continuity restored after 8 weeks Well on Follow up
6	13 F	Intestinal obstruction	Cocoon Interbowel fistulas	Adhesiolysis	Caseating epithelioid cell granulomas	Enterocutaneous fistula Managed conservatively Discharged Well on follow up

The diagnosis of abdominal cocoon was never entertained pre-operatively in any of these cases, and they were all operated upon as cases of acute intestinal obstruction. The operative findings were very much the same in all cases, with partial or complete encasement of the small bowel and omentum by a thin, membranous sac in all, and the cecum and ascending colon in addition in one case. As a rule, there were dense interbowel adhesions in all the cases, and one case also had multiple interbowel fistulae. Gross evidence of tuberculosis such as mesenteric lymphadenopathy, and tubercles over the bowel serosa were present in 4 of the 6 cases.

The post – operative period was not smooth. There was 1 mortality – the patient who was referred to us from outside and was re-explored succumbed in the immediate post-operative period. Of the other 5 cases, 4 developed enterocutaneous fistulae – 3 required re-operation, and the other was managed conservatively. Although all survived, the post-operative period in all was prolonged to an average of 1 month in each of them (range 11 days to 4 months).

A review of the histopathology slides revealed fibrocollagenic tissue with caseating epithelioid cell granulomas with Langhans' and foreign body type of giant cells in 4 cases, but no acid-fast bacilli could be seen. In the patient who was a known case of abdominal Koch's on treatment, only fibrocollagenic tissue with a mixed inflammatory infiltrate could be visualized. The case that was referred to us from outside already had a report of tuberculosis from the tissues biopsied intra-operatively when he came to us, and as such, his slides were not available for review.

## Discussion

The abdominal cocoon remains an uncommon cause of intestinal obstruction, and a search of Medline revealed only about 60 articles dealing with this topic till date. Of these, the majority of cases reported were of the primary type, but the secondary form was also frequently reported, although we could come across only 3 cases with a possible tubercular aetiology – one from the original report by Foo et al [1], another in the report by Sahoo et al [2], and the third, in the report of Lalloo et al [10].

Clinically, these patients with abdominal cocoon present with attacks of colicky pain abdomen, nausea, vomiting with intestinal obstruction that is seldom complete [11]. An abdominal mass may or may not be present [4,9]. Although some authors have described a few radiological signs on plain x-ray, barium series and computerized tomogram scan, it is, as a rule, difficult to be able to make a definite pre – operative diagnosis of this entity [3,6,11]. The diagnosis is usually made at laparotomy, when the encasement of the small bowel within the sac-like cocoon is visualized. Although the disease primarily involves small bowel, it can extend to involve other organs like the large intestine, liver and stomach. The treatment is by lysis of this covering membrane, and rarely, further procedures such as resection, are required [6,7,9,11]. However, these features are probably true of cocoons arising from a non – tubercular etiology. In our experience, the cocoons secondary to tuberculosis can present at any age or sex, and usually present with a complete small bowel obstruction that does not respond to conservative treatment. At surgery, in addition to the covering membrane, there also are dense interbowel adhesions that also need to be freed in order to relieve the obstruction, and hence, the potential for iatrogenic complications is high. Other manifestations of abdominal tuberculosis such as mesenteric abscesses, enlarged and caseating mesenteric lymph nodes, and tubercles over the bowel serosa are also commonly encountered, that may suggest a tubercular etiology.

The histological examination of the membranous tissue in a primary cocoon shows proliferation of fibroconnective tissue with non-specific chronic inflammatory reaction. In our series, the excised membrane showed caseating epithelioid cell granulomas in all but one case – this patient was already on anti-tubercular treatment for previously diagnosed abdominal tuberculosis, thus giving a tubercular aetiology to all our cases. Once the diagnosis of tuberculosis is established these patients need to be put on standard anti-tubercular treatment.
